# Early vs. delayed QTc prolongation in acute poisoning: A prognostic accuracy study—A case series

**DOI:** 10.1371/journal.pone.0309940

**Published:** 2024-09-10

**Authors:** Amirhossein Shahpar, Amirhossein Mirafzal, Mitra Movahedi, Nazanin Zeinali Nezhad

**Affiliations:** 1 Gastroenterology and Hepatology Research Center, Institute of Basic and Clinical Physiology Sciences, Kerman University of Medical Sciences, Kerman, Iran; 2 Department of Emergency Medicine, Kerman University of Medical Sciences, Kerman, Iran; 3 Physiology Research Center, Kerman University of Medical Sciences, Kerman, Iran; Juntendo University: Juntendo Daigaku, JAPAN

## Abstract

Given the limited capacity of intensive care units in many countries, it is crucial to identify reliable prognostic markers to optimize poisoning patients management and improve outcomes. This study aimed to assess the predictive value of three variables, namely the initial QTc interval (iQTc) measured within two hours of admission, the delayed QTc interval (dQTc) measured between 6 and 12 hours of entry, and the QTc interval trend over time (ΔQTc), for mortality in patients with undifferentiated poisoning. A retrospective case series was conducted on 70 patients with undifferentiated poisoning admitted to the intensive care unit (ICU) of Afzalipour Hospital between March 21, 2021, and March 20, 2023. The results of the multivariate analysis revealed that dQTc, base deficit, and creatinine were independently associated with mortality (P value < 0.001). The dQTc had the highest predictive ability, with an area under the curve (AUC) of 0.84, followed by ΔQTc with an AUC of 0.76, and iQTc with an AUC of 0.67. Additionally, the results of the Generalized Estimating Equation model with repeated measurements revealed a higher odds ratio for dQTc (OR, 6.33; 95% CI, 2.54–15.79) compared to iQTc (OR, 4.92; 95% CI, 1.71–14.17). The study concluded that monitoring the dQTc interval could provide valuable prognostic information in acute poisoning cases.

## Introduction

Poisoning is a critical health problem caused by exposure to harmful substances that affect the body [1]. Unintentional poisoning is the primary cause of mortality due to accidental injuries in the United States, followed by motor vehicle accidents [2]. The United States experienced a total of 106,699 fatalities due to drug overdose in 2021. This figure reflects a 14% increase in the age-adjusted mortality rate compared to that in the same period in 2020 [3]. A wide range of visits to emergency departments (EDs) are related to poisoning. It is estimated that out of 843 million ED visits in the United States between 2003 and 2011, nearly 8 million visits (0.9%) were related to poisoning. This number has shown an increasing trend, from 1.8 million visits in 2003–2004 to 2.9 million visits in 2010–2011 [4]. Undifferentiated or unknown poisoned patients pose a significant challenge among admissions with suspected poisoning in the ED. Current recommendations for managing poisoned patients needing emergency cardiovascular care are mainly based on expert consensus and consultations with medical toxicologists or poison control centers [5]. Initial assessments in the ED usually consist of an electrocardiogram (ECG) and laboratory examinations. Healthcare providers must make clinical decisions regarding discharge or hospitalization while considering the medical consequences and potential risks of adverse events [6].

Adverse cardiovascular events (ACVEs) are a significant cause of morbidity and mortality in patients with drug overdose. These events can manifest as myocardial injury, shock, ventricular dysrhythmia, and cardiac arrest [6]. Previous research has demonstrated that the presence of ECG abnormalities, particularly prolonged QTc intervals, increases the risk of ACVE by more than tenfold in patients with suspected acute poisoning [7]. Delayed severe QTc prolongation has been reported in patients receiving opium and selective serotonin reuptake inhibitor (SSRI) overdoses [8, 9]. Electrolyte imbalances, delayed drug absorption, toxicokinetics of the drugs, or drug interactions may lead to delayed QTc (dQTc) prolongation [10].

Even though these cases have been reported, only a few studies have evaluated the potential of initial and delayed QTc (iQTc and dQTc) prolongation and the QTc trend (ΔQTc) as predictors of adverse outcomes in a population of undifferentiated or unknown poisoned patients [10, 11]. In this study, we investigated whether iQTc and dQTc prolongation and ΔQTc in the emergency room could be predictive indicators of the outcome of patients with suspected poisoning, regardless of the type of poisoning. We also aimed to compare the predictive value of each indicator.

## Method

### Study design and time period

This retrospective chart review was conducted to evaluate the predictive value of iQTc and dQTc prolongation and ΔQTc in patients with suspected poisoning admitted to the poisoning intensive care unit (ICU) of Afzalipour Hospital. The study period was from March 21, 2021, to March 20, 2023. The study was granted approval by the Institutional Review Board (IR.KMU.AH.REC.1400.291(. On April 25, 2023, we obtained access to the medical records. Furthermore, throughout the entirety of data access, all information was meticulously anonymized.

### Study setting and population

The data were extracted from the medical records of eligible individuals for this investigation. Patients who met the inclusion criteria were those who had at least two 12-lead ECGs—one within two hours of ED admission and another between 6 and 12 hours of access—and had a confirmed poisoning diagnosis based on urine toxicology, blood toxicology, or a combination of history, physical examination, and circumstantial evidence (e.g., presence of a poison bottle or label discovered by relatives). The exclusion criteria included being under 18 years old; having cardiac disease or comorbid illnesses that could affect survival or outcomes; being transferred from another healthcare facility; having missing ECG, laboratory, or vital signs; using medications known to be associated with QTc prolongation; deaths resulting from causes unrelated to ACVEs; and having an alternative diagnosis.

The study included the adult population (aged ≥ 18 years) who were admitted to the ICU of the hospital with a confirmed diagnosis of acute poisoning within the study period. A wide range of patient information was extracted from the medical records, including demographic data; exposure details (such as the time elapsed from exposure to admission, drug type, and route of exposure); initial mental status assessed using the Glasgow Coma Scale (GCS); vital signs; medical history (including previous drug use and preexisting comorbidity); laboratory results; initial ECGs; repeated ECGs; duration of hospitalization; and outcome. Two independent and trained investigators extracted the relevant information from medical records. Each investigator was blinded to the findings of the other investigator. Any discrepancies in chart abstraction were resolved through discussion.

### Electrocardiographic evaluation

Two physicians performed independent measurements of the QT interval using a standard 12-lead ECG tracing at a 25 mm/s paper speed and 10 mm/mV amplitude. To determine the QT interval, they calculated the mean value derived from at least 3–5 cardiac cycles. This interval was measured from the beginning of the earliest onset of the QRS complex to the end of the T wave. The measurements were taken in leads II, V5, and V6, and the longest value was used. The tangent method was used to measure the QT interval. In this study, the Bazett formula (*QT / √RR*) was used to adjust the QT interval for heart rate. Interobserver agreement was assessed to evaluate the consistency between the two observers. Any significant discrepancies regarding the QTc value were discussed and resolved through consensus and by rechecking the ECG. Only those discrepancies between the observers that led to categorizing a patient in two different categories (prolonged vs normal QTc) were considered significant. If such a discrepancy could not be resolved, the patient was removed from the study. To evaluate the interrater reliability for continuous data, we calculated the concordance correlation coefficient, which was found to be 0.923, indicating excellent agreement. By definition, the QTc interval was considered prolonged if it was equal to or greater than 450 msec. The QTc value calculated within two hours of admission to the ED was labeled the iQTc, while the QTc value calculated between 6 and 12 hours after admission was labeled the dQTc. The difference between dQTc and iQTc (ΔQTc) was then analyzed for trend.

### Exposure and outcome

The primary outcomes in our study were defined as death due to ACVEs. These events were defined as a combination of shock (hypotension requiring vasopressors), myocardial injury (an increase in serum cardiac troponin levels on at least one measurement), ventricular dysrhythmia (ventricular tachycardia or ventricular fibrillation), and cardiac arrest (loss of pulse requiring cardiopulmonary resuscitation). Furthermore, the primary exposure in our study was initial and delayed QTc prolongation. We followed all patients until they were either completely discharged from the hospital units or in-hospital mortality occurred.

### Data analysis

The data are reported as the mean ± standard deviation for continuous variables with a normal distribution. The data are reported as medians with 25th and 75th percentile ranges for continuous variables without a normal distribution. All the data were tested for normality of distribution and equality of standard deviations before analysis. Continuous data were analyzed using Student’s t test and the Mann‒Whitney U test. Categorical data were analyzed using the chi-squared test. Multivariate logistic regression was conducted for all variables significantly correlated with the outcome in the univariate analysis (p value < 0.05). A multivariable logistic regression model was generated using the backward conditional method. To compare the predictive value of iQTc and dQTc as a variable with repeated measurement within patients, we used Generalized Estimating Equations (GEE). GEE is a flexible and widely used statistical method that is effective in analyzing correlated data structures. It was introduced by Liang and Zeger in 1986 and is particularly useful for handling correlated data arising from repeated measures over time [12]. Unlike other models, GEE can accommodate various types of outcome data, including continuous, count, and binary variables. GEE models can also incorporate both time-varying and time-invariant predictors, making them suitable for a wide range of research applications. The GEE model used in this study can be expressed by the following formula: $\sum_{i=1}^{K}\frac{K\partial }{\beta \prime \partial }{Vi}^{-1}(yi-\mu i)=0$, where K is the number of clusters (e.g., subjects), y_i_ is the n_i_ response vector for cluster i., μ_i_ is the n_i_ mean vector for cluster i., V_i_ is the covariance matrix of the response modeled as $Vi={Ai}^{1/2}Ri(\alpha ){Ai}^{1/2}$, A_i_ is a diagonal matrix containing the variances, and R_i_(α) is the working correlation matrix. GEEs can handle longitudinal/clustered data with correlated observations within each cluster or subject. The working correlation structure V_i_ accounts for within-cluster correlation, but it does not need to be specified exactly for GEE estimates to be consistent.

The GEE method is a reliable approach that considers the QTc interval as a within-patients variable with repeated measurements at multiple time points, effectively accounting for the correlated structure of the data. Our GEE model evaluated iQTc and dQTc in predicting mortality by treating QTc interval as prolonged or non-prolonged and timing as a within-subject variable.

We also conducted receiver operating characteristic (ROC) curve analysis for continuous variables that were found to be predictive of the outcome to evaluate their potential ability to predict mortality. Data analysis was performed using IBM SPSS Statistics version 27 for Windows (IBM Corp. Released 2020. IBM SPSS Statistics for Windows, Version 27.0. Armonk, NY: IBM Corp.). The odds ratios (ORs) of each variable were reported with relevant 95% confidence intervals (CIs). P <0.05 was considered to indicate statistical significance.

## Results

### Basic characteristics

**Fig 1:**
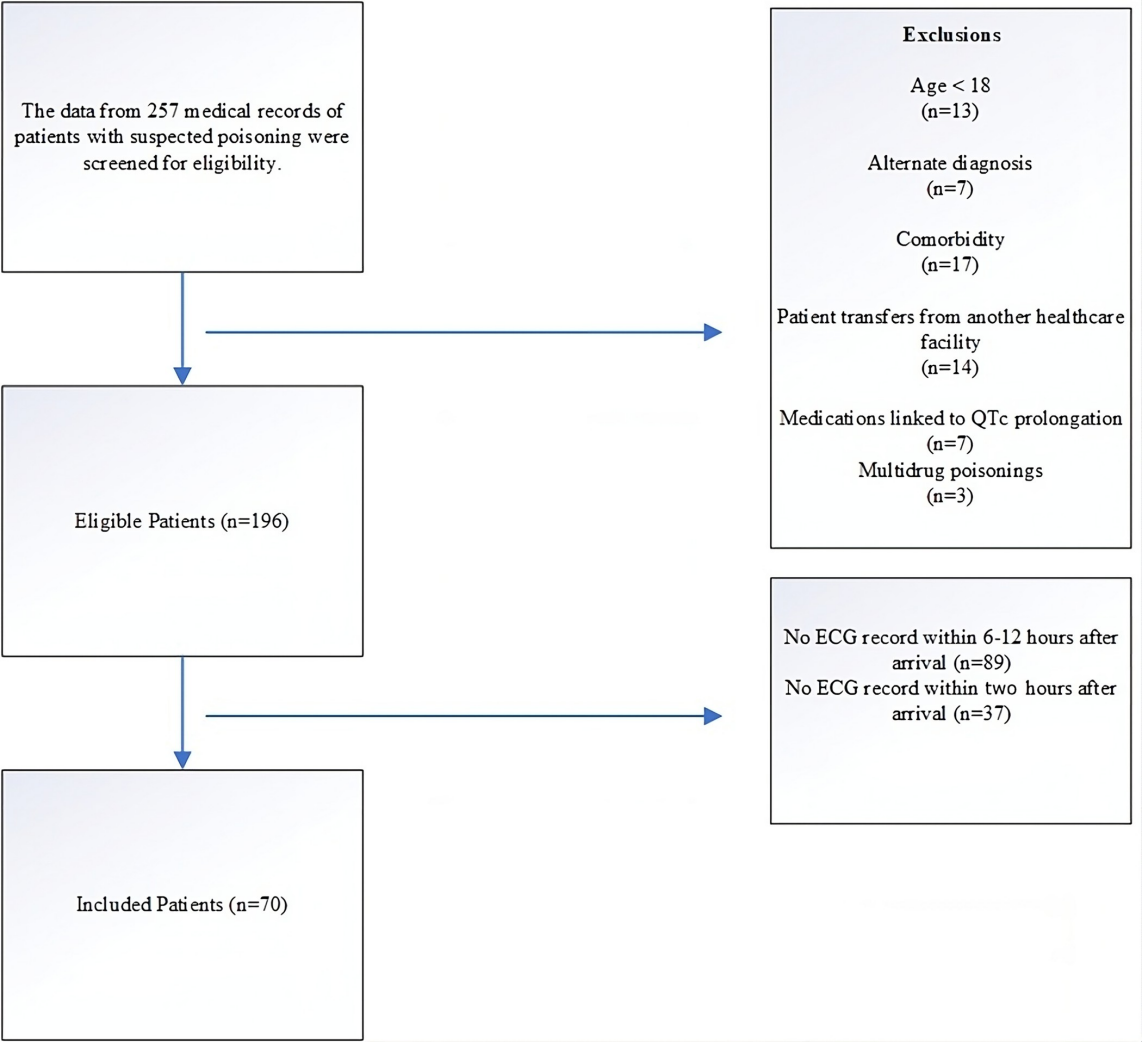
Step-by-step exclusion of patients to determine the study population.

A total of 70 patients were included in the study (Fig 1), 18 (25.7%) were female, and 52 (74.3%) were male. The male-to-female ratio was 2.88. The age range of the whole group of participants was between 18 and 75 years, with a median age of 30.5 years (IQR: 16). The median age of the survivors was 31(IQR:16), and that of the nonsurvivors was 29 (IQR:16). There was no significant difference in age between the survivor and nonsurvivor groups (P value = 0.6). The mortality rate of all patients was 44.3% (31/70), with a male-to-female ratio of 2.44 (22/9). The most common type of drug overdose was benzodiazepines, accounting for 20.0% (14/70) of the total drug use, followed by other drugs. Out of the total number of patients, 26 (37.1%) had a prolonged iQTc, whereas 32 (45.7%) had a prolonged dQTc (Table 1). In our study, it is important to note that all patients were in sinus rhythm upon admission and remained in sinus rhythm during the two ECG recordings for iQTc and dQTc. Table 2 presents the essential characteristics of the quantitative variables. The median duration of hospitalization was three days (IQR: 3). The median time from exposure to admission was 12 hours (IQR: 8.25). The median duration of ICU stay was two days (IQR: 2).

**Table 1:** Distribution of poisoning agents and demographic features among survival status and QTc prolongation.

	Poisoning Agents		All	survivor group Number (%)	non-survivor group Number (%)	Prolonged iQTc	Prolonged dQTc
**Demographic features**	**N (%)**		70 (100%)	39 (55.7%)	31 (44.3%)	26 (37.1%)	32 (45.7%)
	**Age (Median, IQR)**		30.50 (16.00)	31.00 (16.00)	29.00 (16.00)	30.00 (28.00)	29.00 (15.00)
	**Gender, n (%)**	**Men**	52 (74.3%)	30 (76.9%)	22 (71.0%)	17 (64.4%)	19 (59.4%)
**Type of poisoning, n (%)**	**Benzodiazepine**		14 (20.0%)	10 (25.6%)	4 (12.9%)	1 (3.8%)	4 (12.5%)
	**Opioids**		13 (18.6%)	9 (23.1%)	4 (12.9%)	6 (23.1%)	5 (15.6%)
	**CAs**		10 (14.3%)	4 (10.3%)	6 (19.4%)	9 (34.6%)	6 (18.8%)
	**Paraquat**		10 (14.3%)	2 (5.1%)	8 (25.8%)	4 (15.4%)	7 (21.9%)
	**Aluminum. phosphide**		8 (11.4%)	3 (7.7%)	5 (16.1%)	3 (11.5%)	4 (12.5%)
	**Alcohols**		5 (7.1%)	3 (7,7%)	2 (6.5%)	0 (0.0%)	2 (6.3%)
	**Amphetamine**		3 (4.3%)	2 (5.1%)	1 (3.2%)	1 (3.8%)	2 (6.3%)
	**SSRIs**		3 (4.3%)	3 (7.7%)	0 (0.0%)	1 (3.8%)	1 (3.1%)
	**CO**		2 (2.9%)	2 (5.1%)	0 (0.0%)	1 (3.8%)	0 (0.0%)
	**Zinc. phosphide**		2 (2.9%)	1 (2.6%)	1 (3.2%)	0 (0.0%)	1 (3.1%)

CAs: Cyclic Antidepressants, SSRIs: Selective Serotonin Reuptake inhibitors, CO: carbon monoxide, iQTc: QTc at admission, dQTc: QTc 6–12 hours after admission

**Table 2:** Basic statistics for quantitative variables.

	Variables	Mean (SD)	Median (IQR)	Min/Max
Vital Signs^b^	**GCS** ^**a**^	11.45 (3.94)	12.00 (6.00)	3–15
	**HR** ^**a**^	95.45 (19.53)	93.50 (23.75)	57–140
	**SBP (mmHg)**	118.44 (25.87)	-----	60–184
	**DBP (mmHg)** ^**a**^	74.94 (16.05)	75.00 (25.75)	40–122
Laboratory Parameters^b^	**Hb (g/dl)**	14.40 (2.59)	-----	5–20.1
	**Plt (10** ^**9**^ **/L)**	258.34 (89.71)	-----	19–458
	**BD (mmol/L)** ^**a**^	-4.27 (9.07)	-2.8 (11.15)	-29.2–19
	**Na (mEq/L)**	137.90 (4.01)	-----	128–147
	**K (mEq/L)** ^**a**^	4.05 (0.69)	3.90 (0.73)	2.7–6.4
	**Cr (mg/dL)** ^**a**^	1.42 (0.63)	1.20 (1.00)	0.6–3.1
**ECG Parameters**	**iQTc (msec)**	438.42 (37.97)	-----	361–542
	**dQTc (msec)**	450.81 (44.61)	-----	351–588
	**ΔQTc (msec)** ^**a**^	12.38 (40.76)	11.50 (37.00)	-80-145
**Timing Metrics**	**ICU Stay Duration (days)** ^**a**^	3.00 (3.91)	2 (2)	1–24
	**Hospitalization Duration (days)** ^**a**^	4.27 (4.23)	3 (3)	1–24
	**Exposure-to-Admission Delay (hours)** ^**a**^	9.84 (4.93)	12 (8.25)	1–20

HR: heart rate, SBP: systolic blood pressure, DBP: diastolic blood pressure, BD: base deficit, Cr: creatinine, GCS: Glasgow Coma Scale, iQTc: QTc at admission, dQTc: QTc 6–12 hours after admission, ΔQTc: dQTc-iQTc Min/Max: minimum/maximum

^a^ Variables for which medians (IQRs) were reported are non-normally distributed.

^b^ Data were obtained during the first hours of admission.

### Univariate analysis

Concerning the quantitative variables, several variables, including heart rate (P value = 0.02), base deficit (BD) (P value = 0.01), creatinine (P value <0.001), dQTc interval (P value <0.001), ICU Stay Duration **(**P value = 0.005), iQTc interval (P value = 0.03) and ΔQTc (QTc2-QTc1) (P value <0.001), were associated with patient outcome and were significantly greater in the nonsurvival group than in the survival group (Table 3). Nevertheless, due to multiple comparisons, an adjusted analysis was performed to identify variables that had independent associations with the outcome. All variables correlated with outcomes in the univariate analysis were also assessed in the multivariate analysis.

**Table 3:** Associations of quantitative variables with mortality.

	Variables		Mean (SD)		P value
			survivors	Non-survivors	
**Vital Signs**	**Age** ^**a**^		31.00 (16.00)	29.00 (16.00)	0.6
	**Gender, n (%)**	**Male**	30 (76.9)	22 (70.9)	0.38
		**Female**	9 (23.0)	9 (29.0)	
	**GCS** ^**a**^		12.00 (6.00)	15.00 (6.00)	0.23
	**HR** ^**a**^		88.00 (18.00)	100.00 (33.00)	0.02^b^
	**SBP (mmHg)**		116.89 (21.87)	120.38 (30.43)	0.59
	**DBP (mmHg)** ^**a**^		75.00 (25.00)	75.00 (30.00)	0.87
**Laboratory Parameters**	**Hb**		14.47 (1.97)	14.30 (3.24)	0.78
	**Plt**		274.25 (80.90)	238.32 (97.34)	0.10
	**BD (mmol/L)** ^**a**^		0.00 (7.50)	-7.90 (12.90)	0.01^b^
	**Na (mEq/L)**		137.97 (4.11)	137.80 (3.95)	0.86
	**K (mEq/L)** ^**a**^		3.90 (0.80)	4.10 (0.60)	0.28
	**Cr (mg/dL)** ^**a**^		1.10 (0.50)	1.60 (1.20)	<0.001^b^
**ECG Parameters**	**iQTc (msec)**		429.77 (38.23)	449.32 (35.27)	0.03^b^
	**dQTc (msec)**		427.79 (29.49)	479.77 (43.82)	<0.001^b^
	**ΔQTc (msec)** ^**a**^		0.00 (47.00)	25.00 (44.00)	<0.001^b^
**Timing Metrics**	**ICU Stay Duration(days)** ^**a**^		1 (1)	2 (4)	0.005^b^
	**Hospitalization Duration(days)** ^**a**^		3 (3)	3 (4)	0.84
	**Exposure-to-Admission Delay(hours)** ^**a**^		10 (8)	12 (9)	0.52

HR: Heart rate, SBP: systolic blood pressure, DBP: diastolic blood pressure, BD: base deficit, GCS: Glasgow Coma Scale, Cr: creatinine, iQTc: QTc at admission, dQTc: QTc 6–12 hours after admission, ΔQTc: dQTc-iQTc

^a^Variables for which medians (IQRs) are reported are non-normally distributed; the Mann‒Whitney U test was performed for these variables.

^b^Statistically significant

### Multivariate analysis

A multivariable logistic regression model was created using the backward conditional method to identify the most important and independent variables with predictive value for the outcome. The analysis revealed that three variables were independently associated with the outcome. These variables were dQTc (OR, 1.04; 95% CI, 1.01–1.06), BD (OR, 0.91; 95% CI, 0.84–0.99), and creatinine (OR, 6.87; 95% CI, 1.79–26.27) (Table 4).

**Table 4:** Multivariate logistic regression model for predicting mortality.

Variables	Standard Error	Wald	P value	OR (95% CI)
**BD (mmol/L)**	0.04	4.55	0.033	0.91 (0.84–0.99)
**Cr (mg.dL)**	0.68	7.93	0.005	6.87 (1.79–26.27)
**dQTc (msec)**	0.01	11.15	0.001	1.04 (1.01–1.06)

dQTc: QTc 6–12 hours after admission, BD: base deficit, Cr: Creatinine

### Subgroup analysis

To address potential gender-related differences in QTc intervals, we conducted a subgroup analysis focusing solely on male patients (n = 52). This analysis aimed to eliminate the possible confounding effect of gender on our findings. In the male-only univariate analysis, several variables remained associated with patient outcomes in acute poisoning. These included iQTc interval (P value = 0.02), dQTc interval (P value < 0.001), ΔQTc (P value = 0.02), BD (P value < 0.001), and creatinine (P value = 0.002).

Furthermore, we performed a multivariate analysis for the male subgroup. This analysis confirmed that dQTc (OR, 1.03; 95% CI, 1.01–1.06), BD (OR, 0.92; 95% CI, 0.84–1.00), and creatinine (OR, 5.53; 95% CI, 1.36–22.40) continued to be independently associated with the outcome, even after accounting for gender. These results support the robustness of our original findings, suggesting that the prognostic value of QTc interval measurements, particularly dQTc, remains significant even when considering potential gender-related differences in QTc intervals.

It is important to note that our study included only 18 female patients, which represents a relatively small sample size. Due to this limitation, we did not perform a separate subgroup analysis for female patients, as it would likely be underpowered and may not produce reliable or meaningful results.

### Repeated measures analysis

The time between poison exposure and hospital admission varies among patients. This variability can potentially affect the comparison of iQTc and dQTc regarding the outcome. To address this issue, we used the GEE longitudinal model with repeated measures to analyze the relationship between QTc prolongation and the outcome. The results of the GEE analysis revealed that dQTc prolongation (OR, 6.33; 95% CI, 2.54–15.79) is more strongly associated with mortality than iQTc prolongation (OR, 4.92; 95% CI, 1.71–14.17) (Table 5).

**Table 5:** Generalized Estimation Equation (GEE) analysis with repeated measures for predicting mortality.

Variables	Standard Error	Wald	P value	OR (95% CI)
**iQTc Prolongation**	0.53	8.75	0.001	4.92 (1.71–14.17)
**dQTc Prolongation**	0.46	15.70	0.001	6.33 (2.54–15.79)

QTc prolongation: QTc ≥ 440 msec at admission, dQTc prolongation: QTc ≥ 440 msec 6–12 hours after admission

### ROC curve

**Fig 2:**
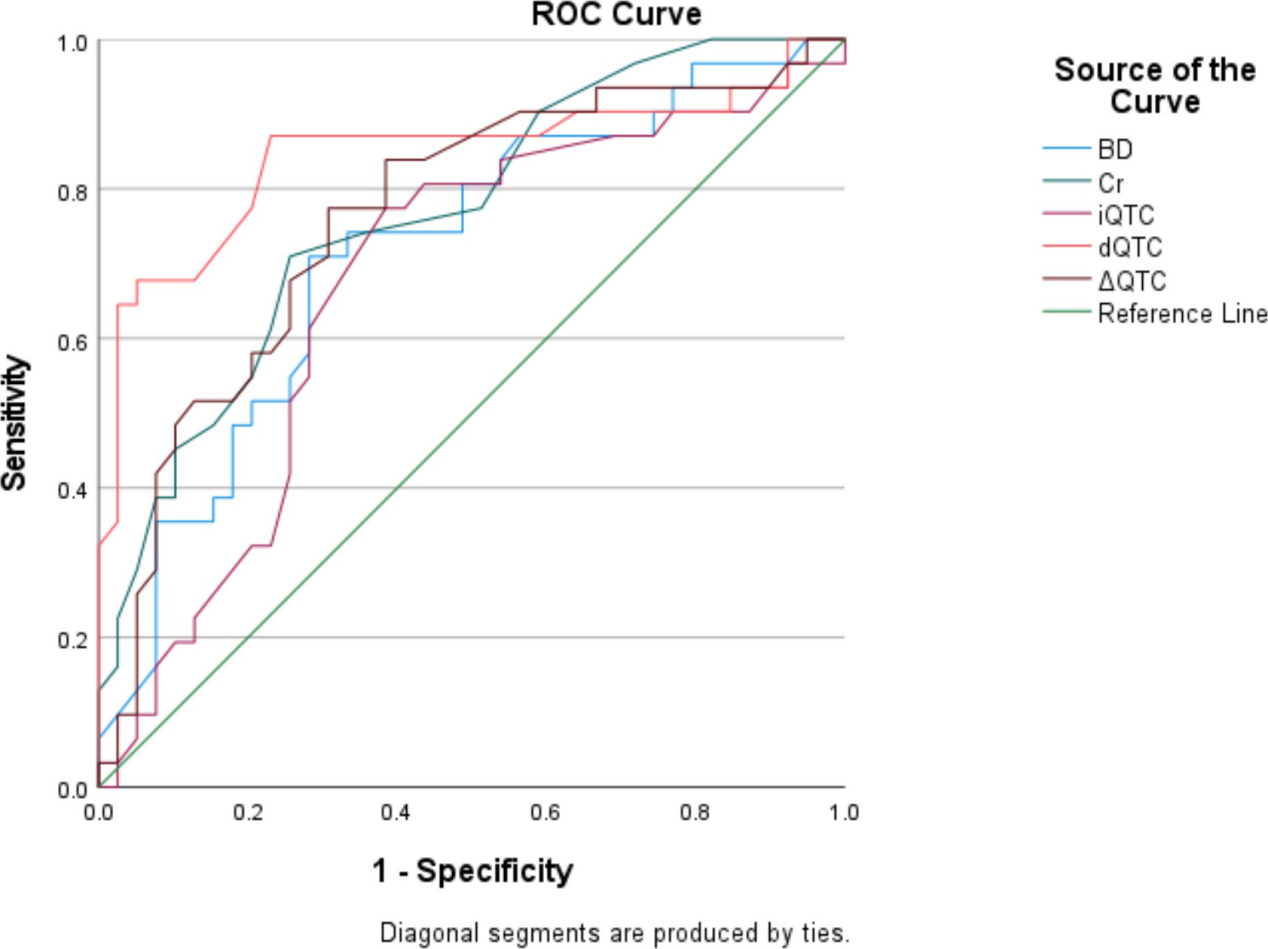
Receiver operating characteristic curve analysis for iQTc, dQTc, ΔQTc, BD, and Cr in predicting mortality. AUC: area under the curve, iQTc: QTc at presentation, dQTc: QTc 6–12 hours after presentation, ΔQTc: dQTc-iQTc, BD: base deficit, Cr: Creatinine.

To assess the accuracy of these variables in predicting mortality, we used receiver operating characteristic (ROC) curves to determine the optimal cutoff point for each variable that maximized sensitivity and specificity. Table 6 provides the specific details of the ROC curve analysis for the variables independently associated with mortality (dQTc, Cr, and BD) and iQTc and ΔQTc (for comparison with dQTc). Among the variables, dQTc had the highest predictive ability, with an area under the curve (AUC) of 0.84 (95% CI, 0.74–0.95). The optimal cutoff point for dQTc was 445 msec, with a sensitivity of 87% and a specificity of 77%. Cr demonstrated an AUC of 0.76 (0.65–0.87). The optimal cutoff point for Cr was 1.25, with a sensitivity of 71% and a specificity of 74%. The AUC for ΔQTc was 0.76 (0.64 to 0.87), demonstrating moderate predictive ability. The optimal cutoff point for ΔQTc was 10.5 msec, with a sensitivity of 77% and a specificity of 70%. The AUC for BD was 0.78 (0.67–0.89). The optimal cutoff point for BD was -3.3, with a sensitivity of 77% and a specificity of 77%. The AUC for iQTc was 0.67 (0.54–0.80). The optimal cutoff point for iQTc was 441 msec, yielding a sensitivity of 77% and a specificity of 62%. These results indicate that, when comparing the ability of iQTc, dQTc, and ΔQTc to predict outcome, dQTc demonstrated the highest ability, followed by ΔQTc and iQTc (Fig 2).

**Table 6:** Receiver Operating Characteristic (ROC) curve for predicting mortality.

Variables	AUC (95%CI)	Standard Error	P value	Cutoff Value	Sensitivity-Specificity (%)
**iQTc (msec)**	0.67 (0.54–0.80)	0.066	0.013	441	77–62
**dQTc (msec)**	0.84 (0.74–0.95)	0.053	<0.001	445	87–77
**ΔQTc (msec)**	0.76 (0.64–0.87)	0.059	<0.001	10.5	77–70
**Creatinine (mg/dL)**	0.76 (0.65–0.87)	0.056	<0.001	1.25	71–74
**Base Deficit (mmol/L)**	0.78 (0.67–0.89)	0.056	<0.001	-3.3	77–77

AUC: area under the curve, iQTc: QTc at presentation, dQTc: QTc 6–12 hours after presentation, ΔQTc: dQTc-iQTc, BD: base deficit, Cr: Creatinine

## Discussion

Poisoning is a primary global health concern, causing an estimated one million yearly morbidities. In some areas, the mortality rate can reach 20%. The World Health Organization predicts that more than 200,000 individuals die annually due to pesticide poisoning alone [13]. In Iran, a developing country with nearly 80 million residents, poisoning accounts for 15% to 20% of emergency department visits. It is one of the most common causes of hospitalization and the second-leading cause of mortality in the country [14].

Several examples of drug poisoning associated with QTc prolongation and mortality have been reported in the literature; these include organophosphates [15], methadone [16], paraquat [17], antidepressants [18], aluminum phosphide [19], lithium [20], carbon monoxide [21], and tramadol [22]. Researchers have also explored the predictive value of QTc prolongation for outcomes in patients with suspected or unknown poisoning. In a large multicenter cohort study in Sweden and Denmark, a prolonged QTc interval (>450 msec in men and >460 msec in women) was associated with a more than threefold increase in 30-day mortality among adult patients with suspected poisoning [11]. Another retrospective observational study of 550 patients requiring toxicology consultation revealed that QTc prolongation (>500 msec) was associated with dysrhythmias and cardiac arrest [23]. A five-year retrospective chart review of patients exposed to substances known to prolong QTc also revealed an association with life-threatening cardiac dysrhythmias [24]. Additionally, a case‒control study involving the prospective identification of 34 cases of acute cardiovascular events in patients suspected of drug poisoning demonstrated an independent association between QTc prolongation and cardiovascular complications [25].

Regarding QTc alteration trends in the ED, while numerous studies have shown associations between the QTc interval and mortality in patients with known or unknown poisoning, only a few studies have compared the predictive value of changes in QTc intervals during admission (QTc trend) with a single measurement or assessed the optimal timing for QTc measurement after admission to maximize its predictive accuracy. Several studies have investigated the dynamic nature of the QTc over admission. One study of 104 patients with carbon monoxide poisoning revealed that the QTc interval tended to increase within the first 24 hours of admission and was correlated with carboxyhemoglobin levels [26]. Additionally, another study has shown increasing changes in QTc intervals over time after administering therapeutic doses of certain drugs that potentially prolong QTc intervals. However, the influence of these alterations on dysrhythmias and mortality was not assessed [27]. Furthermore, in a study of critically ill patients in a general ICU, the QTc intervals gradually increased. This difference was related to azithromycin administration on the third day and high blood creatinine levels on the fifth day [28]. Although this study was not conducted in a poisoning setting, it suggested the dynamic nature of the QTc intervals, indicating the potential need for multiple measurements of QTc during admission. In the ED, these measurements can be performed within 6 to 12 hours, during which critically ill patients are typically transferred to an intensive care setting or a less critical hospital unit.

In our study, we observed a higher mortality rate among females than males (50% vs. 42.3%). This finding contrasts with previous reports that suggested female gender, not using an antidepressant as the method for self-poisoning, and a higher initial GCS score were factors that reduced the risk of a severe or fatal course of self-poisoning [29]. The higher mortality rate in females than males in our study may be attributed to the higher rate of poisoning from cardiotoxic agents, such as Cyclic Antidepressants (CAs), in females (27.7%) compared to males (9.6%). Additionally, the admission GCS in our study was lower among females (mean: 10.05) than males (mean: 11.94). Furthermore, we observed a higher percentage of prolonged QTc intervals (iQTc and dQTc) among females compared to males (iQTc: 50% vs 32.7%, dQTc: 72.2% vs 36.5%).

### Interpretation of findings

In an acute setting of unknown, suspected, or multidrug poisoning, it would be practically impossible to determine the effects of probable agents and evaluate the risk for QTc prolongation. However, performing QTc measurements at regular intervals, such as two measurements at 6-hour intervals, can help overcome such limitations, make a more precise prediction of adverse outcomes, and reassess the disposition of patients, even in a busy ED. Our findings suggest that QTc measurements taken 6–12 hours after admission are more accurate predictors of in-hospital mortality than are the initial QTc measurements taken upon admission or QTc trends during the same period. Moreover, the use of QTc measurements after 6–12 hours of admission may be helpful for re-evaluating and confirming (or perhaps altering) the initial predictions. However, developing an outcome prediction model or scoring system within a few hours of admission would be more attractive. Additionally, based on our results, even though a threshold of 450–460 msec is generally recommended for the definition of QTc prolongation and a range of 480–500 msec is mostly considered high risk, considering that a lower cutoff point (440 msec) may be of greater predictive value. However, the heterogeneity of the data on this issue in the literature may confuse practitioners. We also noted that the time lapse between poison exposure and hospital admission varies among patients and may impact the true prognostic significance of QTc when evaluated without taking this time gap into account. While we acknowledge the challenges of accurately determining the exact time of exposure in emergency situations, we strongly recommend further investigations that consider the timing of iQTc or dQTc from the moment of exposure rather than admission to enhance the reliability of the results.

### Limitations

Our study has various methodological limitations. First, it was a retrospective chart review with a restricted sample size due to time and human resource limitations. Most of the diagnoses were made based on medical history and qualitative urine dipstick tests, which have high rates of false positive and false negative results. Using the Bazett formula for calculating QTc could also be a source of limitations, as it tends to underestimate QTc at heart rates less than 60 and overestimate it at heart rates greater than 120. The retrospective nature of the data collection limits our ability to perform more frequent measurements and more precise timing of ECGs in the ED. Additionally, the sample size of female patients was relatively small (n = 18), which limited our ability to perform a separate subgroup analysis for females. This imbalance in gender distribution may affect the generalizability of our findings across genders. A prospective study involving more patients is needed to overcome these limitations.

## Conclusion

In conclusion, the predictive value of the QTc interval for mortality in acute poisoning patients may be greater when assessing the QTc interval after 6–12 hours of admission than when measuring the QTc at entry or tracking changes in the QTc between these two time points. We suggest that physicians reassess the QTc interval after 6–12 hours of initial evaluation and incorporate this information into risk stratification scores for acute poisoning. Furthermore, setting a lower limit for QTc interval prolongation may be advantageous for identifying patients at high risk of acute poisoning.

## Supporting information

S1 Data (XLSX)

## References

[1] Haddad LM, Winchester JF, Shannon MW, Borron SW, Burns MJ. Haddad and Winchester’s clinical management of poisoning and drug overdose. (No Title). 2007.

[2] Centers for Disease Control and Prevention. Injuries and violence are leading causes of death.2023. https://www.cdc.gov/injury/wisqars/animated-leading-causes.html. Accessed 21 Sept 2023.

[3] Spencer MR, Miniño AM, Warner M. Drug overdose deaths in the United States, 2001–2021. NCHS data brief. 2022 Dec 1;457:1–8. 36598401

[4] Mazer-Amirshahi M, Sun C, Mullins P, Perrone J, Nelson L, Pines JM. Trends in Emergency Department Resource Utilization for Poisoning-Related Visits, 2003–2011. J Med Toxicol. 2016;12: 248–254. doi: 10.1007/s13181-016-0564-6 27342464 PMC4996794

[5] Parris MA, Calello DP. Found down: approach to the patient with an unknown poisoning. Emerg Med Clin. 2022;40: 193–222. 10.1016/j.emc.2022.01.011 35461619

[6] Manini AF, Hoffman RS, Stimmel B, Vlahov D. Clinical Risk Factors for In‐hospital Adverse Cardiovascular Events After Acute Drug Overdose. Bird S, editor. Acad Emerg Med. 2015;22: 499–507. doi: 10.1111/acem.12658 25903997 PMC4426077

[7] Manini AF, Nair AP, Vedanthan R, Vlahov D, Hoffman RS. Validation of the Prognostic Utility of the Electrocardiogram for Acute Drug Overdose. J Am Heart Assoc. 2017;6: e004320. doi: 10.1161/JAHA.116.004320 28159815 PMC5523748

[8] Beach SR, Celano CM, Sugrue AM, Adams C, Ackerman MJ, Noseworthy PA, et al. QT prolongation, torsades de pointes, and psychotropic medications: a 5-year update. Psychosomatics. 2018;59: 105–122. doi: 10.1016/j.psym.2017.10.009 29275963

[9] Martell BA, Arnsten JH, Krantz MJ, Gourevitch MN. Impact of methadone treatment on cardiac repolarization and conduction in opioid users. Am J Cardiol. 2005;95: 915–918. doi: 10.1016/j.amjcard.2004.11.055 15781034

[10] Shastry S, Aluise ER, Richardson LD, Vedanthan R, Manini AF. Delayed QT Prolongation: Derivation of a Novel Risk Factor for Adverse Cardiovascular Events from Acute Drug Overdose. J Med Toxicol. 2021;17: 363–371. doi: 10.1007/s13181-021-00855-2 34449039 PMC8455785

[11] Hansen CS, Pottegård A, Ekelund U, Jensen HK, Forberg JL, Brabrand M, et al. Association between QTc prolongation and mortality in patients with suspected poisoning in the emergency department: a transnational propensity score matched cohort study. BMJ Open. 2018;8. Available: https://www.ncbi.nlm.nih.gov/pmc/articles/PMC6042584/10.1136/bmjopen-2017-020036 PMC6042584 29982199

[12] LIANG K-Y, ZEGER SL. Longitudinal data analysis using generalized linear models. Biometrika. 1986;73: 13–22. doi: 10.1093/biomet/73.1.13

[13] Shadnia S, Esmaily H, Sasanian G, Pajoumand A, Hassanian-Moghaddam H, Abdollahi M. Pattern of acute poisoning in Tehran-Iran in 2003. Hum Exp Toxicol. 2007;26: 753–756. doi: 10.1177/0960327107083017 17984147

[14] Alinejad S, Zamani N, Abdollahi M, Mehrpour O. A Narrative Review of Acute Adult Poisoning in Iran. Iran J Med Sci. 2017;42: 327–346. doi: 10.1177/0960327110361501 28761199 PMC5523040

[15] Liu S-H, Lin J-L, Weng C-H, Yang H-Y, Hsu C-W, Chen K-H, et al. Heart rate-corrected QT interval helps predict mortality after intentional organophosphate poisoning. PLoS One. 2012;7: e36576. doi: 10.1371/journal.pone.0036576 22574184 PMC3344908

[16] Farsi D, Mirafzal A, Hassanian-Moghaddam H, Azizi Z, Jamshidnejad N, Zehtabchi S. The Correlation Between Prolonged Corrected QT Interval with the Frequency of Respiratory Arrest, Endotracheal Intubation, and Mortality in Acute Methadone Overdose. Cardiovasc Toxicol. 2014;14: 358–367. doi: 10.1007/s12012-014-9259-x 24811951

[17] Lin C-C, Hsu K-H, Shih C-P, Chang G-J. Hemodynamic and electromechanical effects of paraquat in rat heart. Plos One. 2021;16: e0234591. doi: 10.1371/journal.pone.0234591 33793552 PMC8016255

[18] Açikalin A, Satar S, Avc A, Topal M, Kuvandk G, Sebe A. QTc intervals in drug poisoning patients with tricyclic antidepressants and selective serotonin reuptake inhibitors. Am J Ther. 2010;17: 30–33. doi: 10.1097/MJT.0b013e318197eec6 19417591

[19] Soltaninejad K, Beyranvand M-R, Momenzadeh S-A, Shadnia S. Electrocardiographic findings and cardiac manifestations in acute aluminum phosphide poisoning. J Forensic Leg Med. 2012;19: 291–293. doi: 10.1016/j.jflm.2012.02.005 22687771

[20] Truedson P, Ott M, Lindmark K, Ström M, Maripuu M, Lundqvist R, et al. Effects of Toxic Lithium Levels on ECG—Findings from the LiSIE Retrospective Cohort Study. J Clin Med. 2022;11: 5941. doi: 10.3390/jcm11195941 36233807 PMC9572509

[21] Wang Y-M, Huang C-C, Liu K-F, Chou C-L, Lee J-T, Hung S-Y, et al. Exercise-induced myocardial ischemia presenting as exercise intolerance after carbon monoxide intoxication and smoke inhalation Injury: case report. BMC Cardiovasc Disord. 2022;22: 570. doi: 10.1186/s12872-022-03019-4 36575398 PMC9795777

[22] Manouchehri A, Nekoukar Z, Malakian A, Zakariaei Z. Tramadol poisoning and its management and complications: a scoping review. Ann Med Surg 2012. 2023;85: 3982–3989. doi: 10.1097/MS9.0000000000001075 37554850 PMC10406095

[23] Robison LB, Brady WJ, Robison RA, Bracy C, Schneck M, Charlton N. QT interval prolongation and the rate of malignant ventricular dysrhythmia and cardiac arrest in adult poisoned patients. Am J Emerg Med. 2021;46: 156–159. doi: 10.1016/j.ajem.2021.04.077 33957571

[24] Ryan K, Benz P, Zosel A, Farkas A, Theobald J. QTc Prolongation in Poison Center Exposures to CredibleMeds List of Substances with “Known Risk of Torsades de Pointes.” Cardiovasc Toxicol. 2022;22: 866–877. doi: 10.1007/s12012-022-09764-4 35930218

[25] Manini AF, Nelson LS, Skolnick AH, Slater W, Hoffman RS. Electrocardiographic Predictors of Adverse Cardiovascular Events in Suspected Poisoning. J Med Toxicol. 2010;6: 106–115. doi: 10.1007/s13181-010-0074-x 20361362 PMC3550283

[26] Yelken B, Tanrıverdi B, Çetinbaş F, Memiş D, Süt N. The assessment of QT intervals in acute carbon monoxide poisoning. Anatol J Cardiol Kardiyol Derg. 2009;9. Available: https://www.acarindex.com/pdfler/acarindex-9969a37af1feb1c81b9edfd179156200.pdf 19819791

[27] Vieweg WVR, Hasnain M, Howland RH, Hettema JM, Kogut C, Wood MA, et al. Citalopram, QTc interval prolongation, and torsade de pointes. How should we apply the recent FDA ruling? Am J Med. 2012;125: 859–868. doi: 10.1016/j.amjmed.2011.12.002 22748401

[28] Farzanegan B, Hosseinpoor Z, Baniasadi S, Seyyedi SR, Rajabi M. An Observational Study of QTc Prolongation in Critically Ill Patients: Identification of Incidence and Predictors. Indian J Crit Care Med Peer-Rev Off Publ Indian Soc Crit Care Med. 2020;24: 270–275. doi: 10.5005/jp-journals-10071-23411 32565638 PMC7297246

[29] Geith S, Lumpe M, Schurr J, Rabe C, Ott A, Zellner T, et al. Characteristics and predictive factors of severe or fatal suicide outcome in patients hospitalized due to deliberate self-poisoning. PLOS ONE. 2022;17: e0276000. doi: 10.1371/journal.pone.0276000 36327226 PMC9632874

